# Carney Complex: case report and review

**DOI:** 10.1186/1749-8090-6-25

**Published:** 2011-03-05

**Authors:** Shirish S Borkar, Sevagur G Kamath, Nitin Kashyap, Sunil CV Sagar, Lakshmi Rao, Raj Warrier, Aman Chauhan

**Affiliations:** 1 Department of Cardiovascular and thoracic surgery, Kasturba Medical College, Manipal University, Manipal, India; 2 Department of Cardiac Anaesthesia, Kasturba Medical College, Manipal University, Manipal, India; 3 Department of Pathology, Kasturba Medical College, Manipal University, India; 4 Department of Hemato-oncology, Kasturba Medical College, Manipal University, India; 5 Intern, Kasturba Medical College, Manipal University, India

## Abstract

Carney complex is a very rare multiple neoplasia syndrome with cardiac, cutaneous, and neural tumours with a variety of pigmented lesion of skin. We are reporting a rare case of carney complex in which left atrial myxoma with superficial angiomyxoma, giant cell tumour of bone and lentigines showed a unique association. This patient underwent successful surgical excision of left atrial myxoma under cardiopulmonary bypass.

## Introduction

Carney complex is a rare syndrome characterized by neoplasia involving heart, central nervous system and endocrine organs. Presence of pigmented skin and mucosal lesions are an important hallmark of this syndrome. We report a case of left atrial myxoma with giant cell tumor, superficial angiomyxoma and skin pigmentation.

## Case report

A 47 year old male with one year history of exertional dyspnea & palpitation was admitted with complaints of syncopal attacks of sudden onset. He denied any head ache, chest pain, palpitation, edema legs, persistent fever, weight loss or loss of appetite.

**Figure 1:**
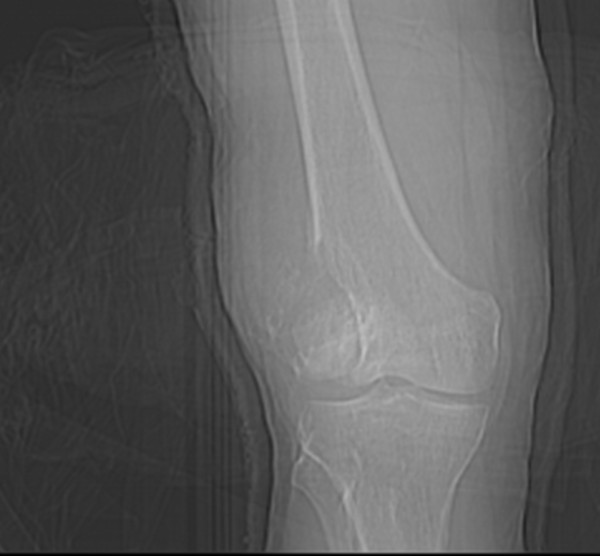
**X-Ray lower end of femur showing Giant Cell Tumor**.

**Figure 2:**
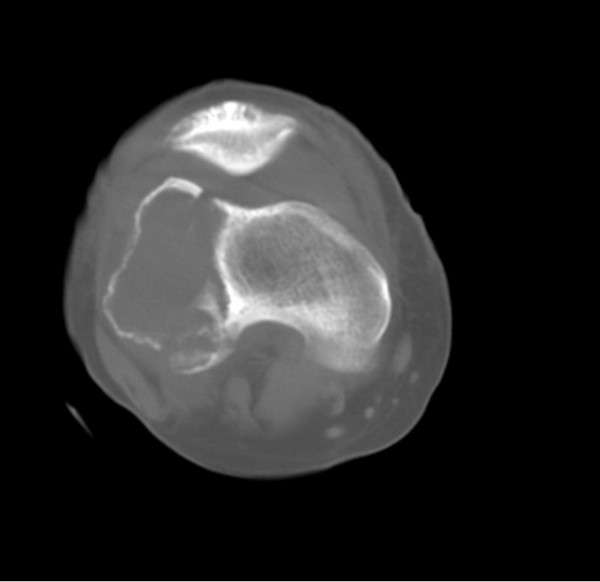
**CT Scan of Knee Joint showing Tumor extent**.

He had been seen at the trauma center 4 years ago following a bike accident. X-Ray at that time showed a lytic lesion in the distal end of the right femur with destruction of the lateral cortex and break in the anterior and posterior cortex of the lateral condyle with extension of the lesion to the distal femoral articular surface and a pathological fracture and periosteal reaction along the lateral aspect of distal femur (Figure 1). CT scan of right lower femur showed expansile lytic lesion noted in the lateral condyle of femur with associated soft tissue mass noted extending out side the cortical margins with calcific densities and bony fragments within (Figure 2).

**Figure 3:**
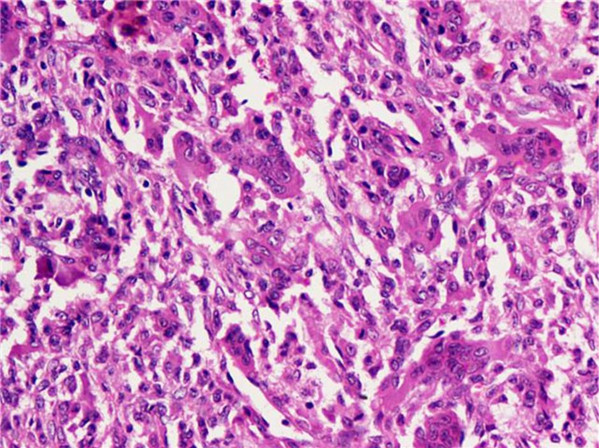
**Giant cell tumor**. Sheets of mononuclear stromal cells with scattered multinucleated osteoclast-like tumor giant cells.

A biopsy was sent from lytic lesion of fractured lower end of femur. A curettage followed by cement application with cancellous leg screw was done. After 15 days bone cement removal, bone grafting and internal fixation with condylar blade plate was performed. Post operatively patient had minimal restriction of right knee flexion. Clinical and radiological follow up showed healing of the lesion. Cut section of the curettage material consisted of multiple hemorrhagic and tiny grey white focal areas. Microscopy showed bony trabeculae and a tumour composed of sheets of mononuclear stromal cells and many scattered multi nucleated osteoclast like giant cells (Figure 3). Histologic diagnosis was Giant cell tumor of the bone.

**Figure 4:**
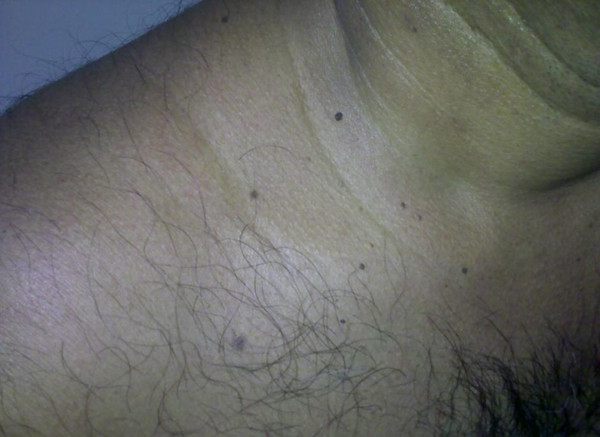
**Skin Pigmentation**.

**Figure 5:**
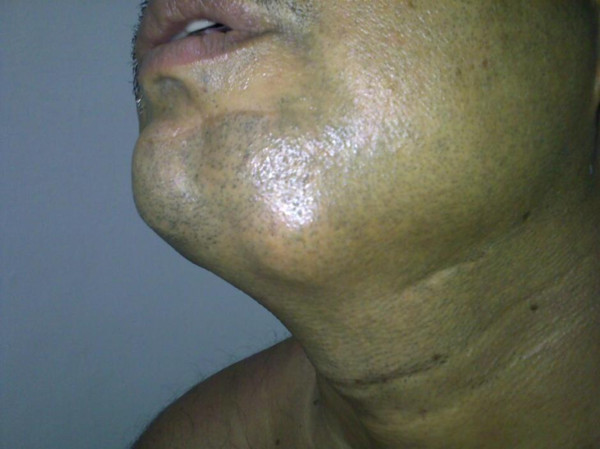
**Superficial Angiomyxoma **. A small swelling in the submandibular region.

**Figure 6:**
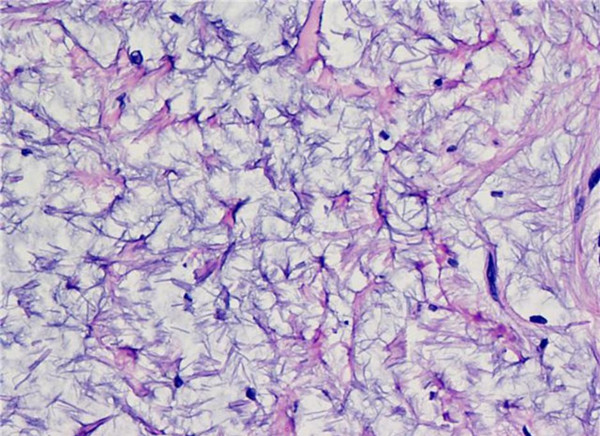
**Superficial angiomyxoma**. Spindle to stellate fibroblasts in myxoid stroma with thin walled capillaries.

He had multiple spotty pigmentations (lentigines) on his trunk for several years (Figure 4). He also had been noted to have swelling of submandibular region requiring three times surgical excisions for the recurrence of the mass (Figure 5) which was pathologically confirmed to be a poorly circumscribed lesion composed of myxoid nodule containing thin walled capillaries along with spindle shaped or stellate fibroblasts. The features were suggestive of superficial angiomyxoma (Figure 6).

**Figure 7:**
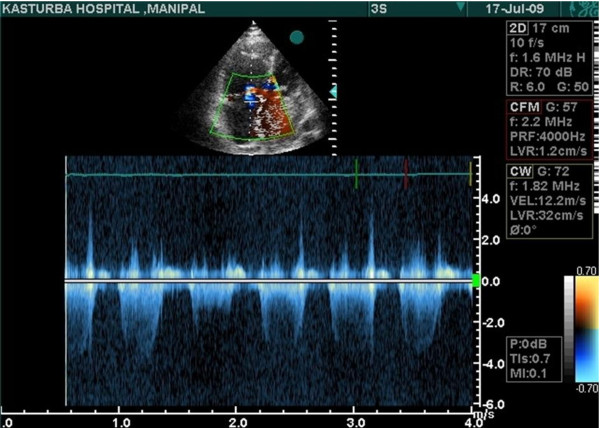
**An intra-cardiac tumour attached to inter atrial septum, which was almost filling the left atrium & obstructing the mitral inflow**.

His cardiac exam showed normal sinus rhythm at 84 beats per minutes and blood pressure of 130/80 mmHg. Mitral first heart sound was slightly accentuated, but the pulmonic sound was normal. Grade-I diastolic murmur was heard over the mitral area. Opening snap was absent. Lungs were clear and chest radiograph showed slight cardiomegaly. Trans-thoracic Echo cardiography revealed an intra-cardiac tumour attached to inter atrial septum, which was almost filling the left atrium & obstructing the mitral inflow. Moderate amount of mitral regurgitation was present (Figure 7). Coronary angiogram showed normal epicardial coronary arteries.

Patient underwent surgical excision of left atrial myxoma under cardiopulmonary bypass through right atrial approach. When a finger was introduced through the right atrial appendage a firm, smooth, egg sized tumour was encountered. The mitral valve was normal in structure and function. It was excised under vision. The patient had an uneventful recovery without neurologic or renal damage with significant relief of clinical symptoms. Follow up Echocardiography after 6 months showed no evidence of any intra cardiac recurrence.

**Figure 8:**
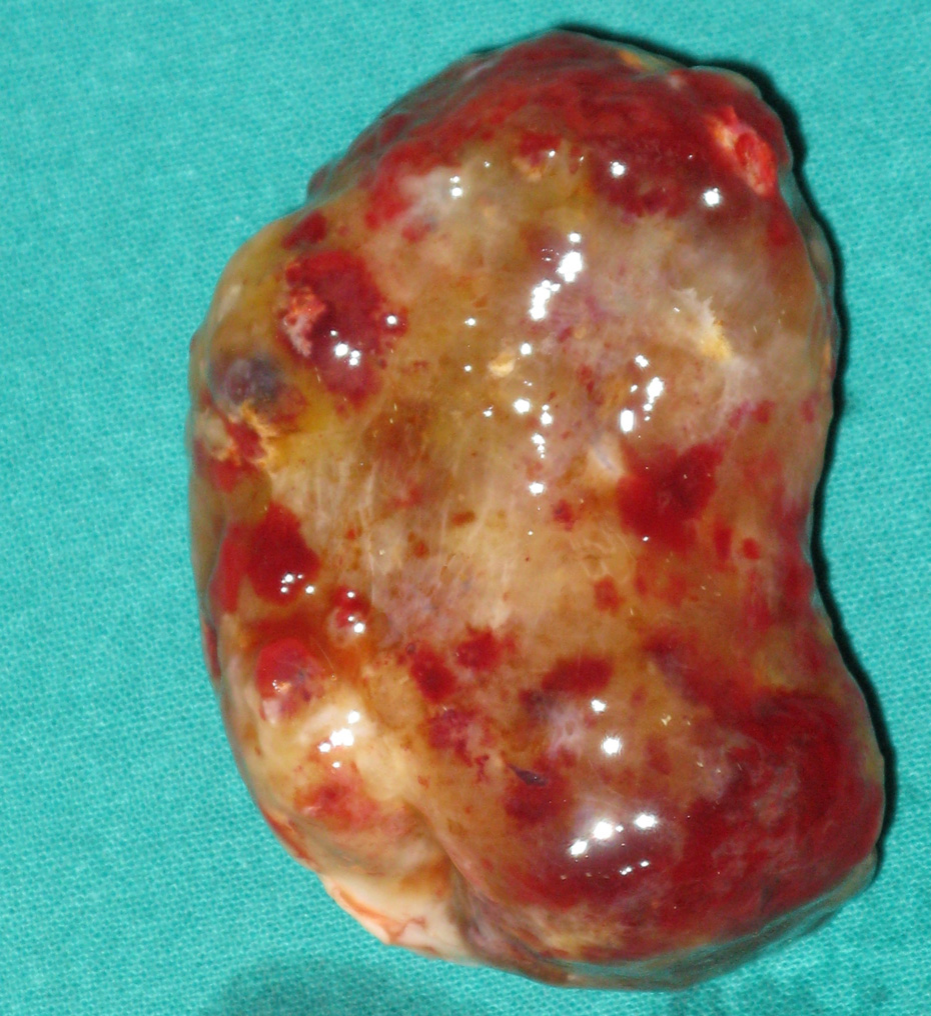
**Gross picture of resected atrial myxoma showing glistening myxoid appearance with areas of congestion**.

Pathological examination of the tumour revealed a solitary mass weighing 50 gms & measuring 6.5 × 4.5 × 2.5 cms. Externally the tumour appeared congested, shining with myxoid areas (Figure 8).

**Figure 9:**
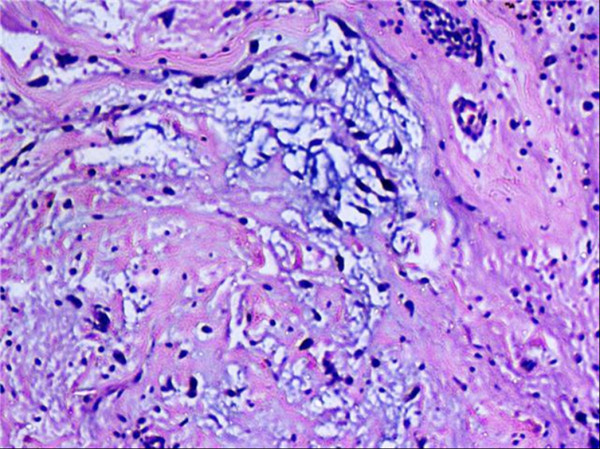
**Atrial myxoma**. Small polygonal to spindle shaped cells in myxoid stroma along with focal lymphoplasmacytic infiltrate.

Microscopically it was a hypocellular myxoid tumour with small polygonal, spindle & ovoid tumour cells (Myxoid cells) with round to oval nucleus, scanty eosinophilic cytoplasm, arranged in strands, along with large " Lipidic" cells having abundant vacuolated, clear cytoplasm arranged around thin walled blood vessels in perivascular pattern. Focal nesting of tumour cells was seen. Stroma showed extensive myxoid change. Hemosiderin laden macrophage, focal dense lymphocytic infiltrate, plasma cells, mast cells were also seen (Figure 9).

## Discussion

Carney complex as described by J. Aiden Carney in 1985 is an autosomal dominant disorder characterized by neoplasia involving heart, central nervous system and endocrine organs. Presence of pigmented skin and mucosal lesions along with these tumors is an important hallmark of this syndrome [1,2]. Most cases are familial and the median age of presentation is 20 years. Carney's complex can manifest itself as spotty cutaneous pigmentation, cutaneous myxomas, cardiac myxomas, psammomatous melanotic schwanoma (PMS), acromegaly, large cell calcifying sertoli cell tumour (LCCSCT), thyroid carcinoma or nodule & breast adenoma.

The most recent diagnostic criteria for Carney Complex includes clinical findings such as spotty skin pigmentation, cutaneous and cardiac myxomas, breast myxomatosis, paradoxical positive response of urinary gluco- corticosteroids to dexamethasone administration during liddle's test, acromegaly, Blue nevus, epithelioid blue nevus, osteochondromyxoma, thyroid carcinoma and mutation of the PRKARIA gene etc. [1].

Lentigines & blue nevi followed by cardiac myxomas are the most common clinical manifestations of the complex. Cardiac myxomas show no age, sex or location preponderance and they are notorious for frequent recurrences [3]. Approximately 7% of all cardiomyxomas occur in association with Carney's complex. Fever, joint pain, palpitations, diastolic murmur in the mitral area and a "tumor plop" is often associated with Carney's complex.

Echo Cardiography can accurately determine the location, size, shape, attachment and mobility of the tumour. Coronary angiography is only advised if coronary artery disease is suspected or if the patient is above 40 years of age [4]. Surgical resection is the treatment of choice and should be pursued immediately once the diagnosis is confirmed [5].

Histologically, superficial angiomyxoma is characterized by a myxoid lesion with prominent thin walled blood vessels. The usual site for angiomyxoma is dermis and cutis especially in head and neck region [6]. Incomplete excision of superficial angiomyxoma is documented to have high recurrence rates [7].

Among bone tumours, osteochondromyxoma has been described in literature which affects about 1% patients of Carney complex. These tumors present as painless mass in diaphyses of distal long bones as well as small flat bones. In this case, we report the presence of giant cell tumour in association with Carney complex. This is the first case report of such an association. Complete tumor resection is curative whereas incomplete resection is associated with high rates of recurrence [8].

At molecular level mutations in chromosome 2 in band p16 and chromosome 17 in bands q22-24 are associated with Carney complex. PRKAR1alpha is a tumor suppressor gene which is found to be mutated in almost 50% of carney complex cases [9].

## Conclusion

This case is the first reported case of Left atrial myxoma, lentigines, superficial angiomyxoma and giant cell tumour of bone in a middle aged patient.

## Consent

Written and informed consent obtained for publication of this case report and accompanying images. A copy of the written consent is available for review by editor- in- chief of this journal

## Competing interests

The authors declare that they have no competing interests.

## Authors' contributions

SB and SG were involved in the surgery and Patient care. NK and AC wrote the manuscript. RW and SS supervised the manuscript. LR provided pathological opinion. All authors read and approved the final manuscript.
